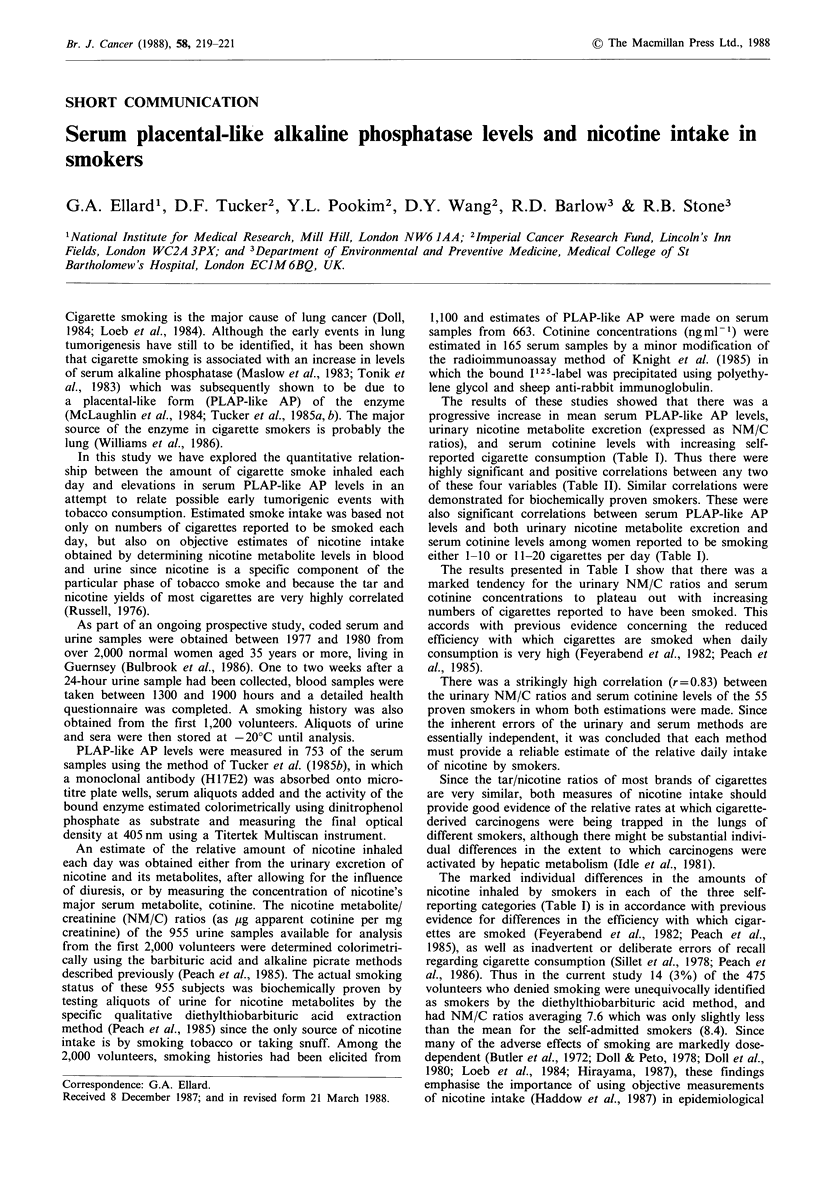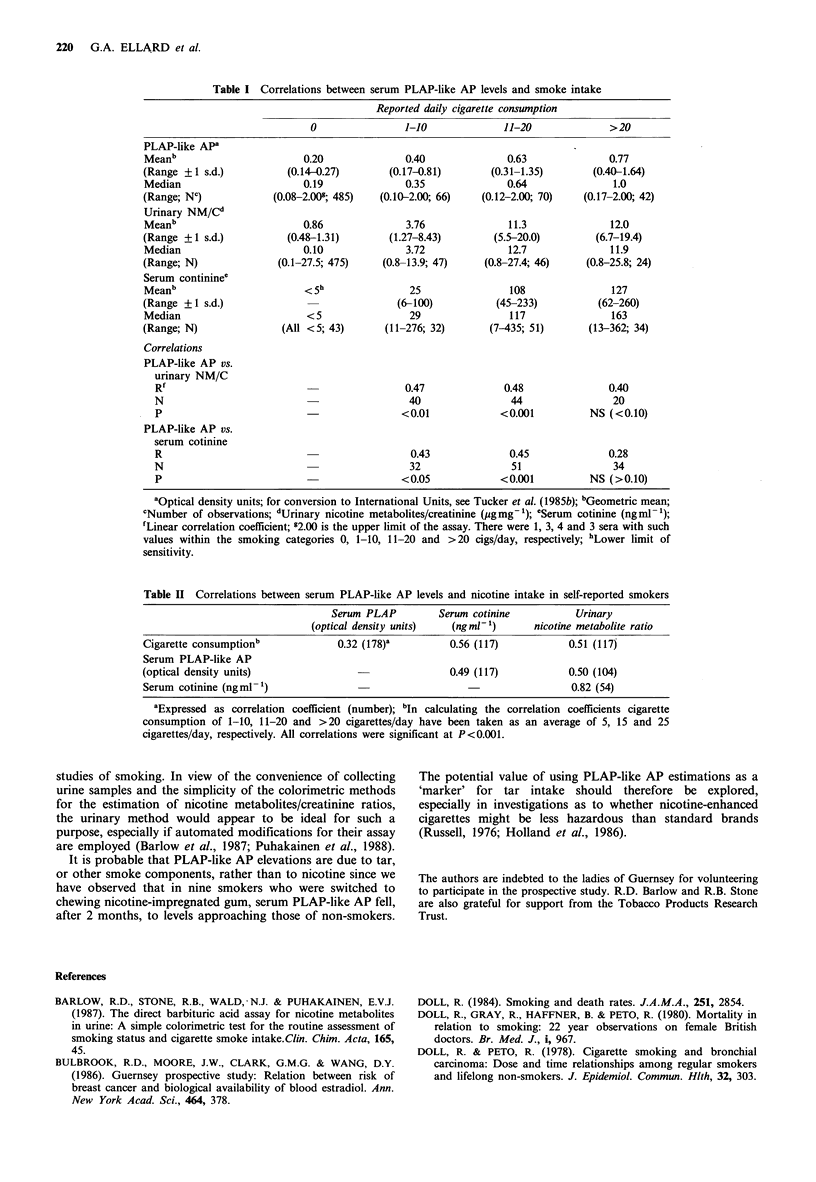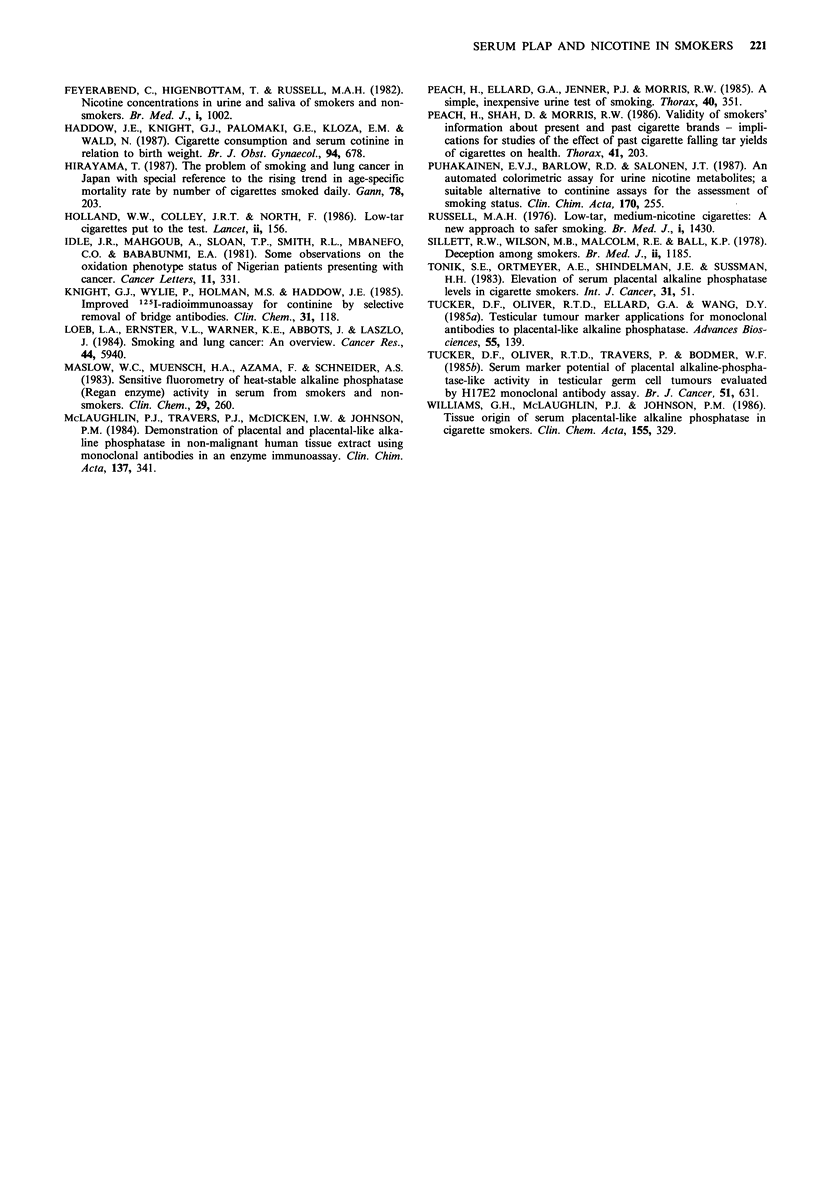# Serum placental-like alkaline phosphatase levels and nicotine intake in smokers.

**DOI:** 10.1038/bjc.1988.198

**Published:** 1988-08

**Authors:** G. A. Ellard, D. F. Tucker, Y. L. Pookim, D. Y. Wang, R. D. Barlow, R. B. Stone

**Affiliations:** National Institute for Medical Research, Mill Hill, London, UK.


Br. J. Cancer (1988), 58, 219-221 © The Macmillan Press Ltd., 1988

SHORT COMMUNICATION

### Serum placental-like alkaline phosphatase levels and nicotine intake in smokers

G.A. Ellard[1], D.F. Tucker[2], Y.L. Pookim[2], D.Y. Wang[2], R.D. Barlow[3] & R.B. Stone[3]

[1]National Institute for Medical Research, Mill Hill, London NW6 1AA; [2]Imperial Cancer Research Fund, Lincoln's Inn Fields, London WC2A 3PX; and [3]Department of Environmental and Preventive Medicine, Medical College of St Bartholomew's Hospital, London EC1M 6BQ, UK.

Cigarette smoking is the major cause of lung cancer (Doll, 1984; Loeb *et al.*, 1984). Although the early events in lung tumorigenesis have still to be identified, it has been shown that cigarette smoking is associated with an increase in levels of serum alkaline phosphatase (Maslow *et al.*, 1983; Tonik *et al.*, 1983) which was subsequently shown to be due to a placental-like form (PLAP-like AP) of the enzyme (McLaughlin *et al.*, 1984; Tucker *et al.*, 1985*a*, *b*). The major source of the enzyme in cigarette smokers is probably the lung (Williams *et al.*, 1986).

In this study we have explored the quantitative relationship between the amount of cigarette smoke inhaled each day and elevations in serum PLAP-like AP levels in an attempt to relate possible early tumorigenic events with tobacco consumption. Estimated smoke intake was based not only on numbers of cigarettes reported to be smoked each day, but also on objective estimates of nicotine intake obtained by determining nicotine metabolite levels in blood and urine since nicotine is a specific component of the particular phase of tobacco smoke and because the tar and nicotine yields of most cigarettes are very highly correlated (Russell, 1976).

As part of an ongoing prospective study, coded serum and urine samples were obtained between 1977 and 1980 from over 2,000 normal women aged 35 years or more, living in Guernsey (Bulbrook *et al.*, 1986). One to two weeks after a 24-hour urine sample had been collected, blood samples were taken between 1300 and 1900 hours and a detailed health questionnaire was completed. A smoking history was also obtained from the first 1,200 volunteers. Aliquots of urine and sera were then stored at $-20°C$ until analysis.

PLAP-like AP levels were measured in 753 of the serum samples using the method of Tucker *et al.* (1985*b*), in which a monoclonal antibody (H17E2) was absorbed onto microtitre plate wells, serum aliquots added and the activity of the bound enzyme estimated colorimetrically using dinitrophenol phosphate as substrate and measuring the final optical density at 405 nm using a Titertek Multiscan instrument.

An estimate of the relative amount of nicotine inhaled each day was obtained either from the urinary excretion of nicotine and its metabolites, after allowing for the influence of diuresis, or by measuring the concentration of nicotine's major serum metabolite, cotinine. The nicotine metabolite/creatinine (NM/C) ratios (as $\mu$g apparent cotinine per mg creatinine) of the 955 urine samples available for analysis from the first 2,000 volunteers were determined colorimetrically using the barbituric acid and alkaline picrate methods described previously (Peach *et al.*, 1985). The actual smoking status of these 955 subjects was biochemically proven by testing aliquots of urine for nicotine metabolites by the specific qualitative diethylthiobarbituric acid extraction method (Peach *et al.*, 1985) since the only source of nicotine intake is by smoking tobacco or taking snuff. Among the 2,000 volunteers, smoking histories had been elicited from 1,100 and estimates of PLAP-like AP were made on serum samples from 663. Cotinine concentrations ($ng\,ml^{-1}$) were estimated in 165 serum samples by a minor modification of the radioimmunoassay method of Knight *et al.* (1985) in which the bound $I^{125}$-label was precipitated using polyethylene glycol and sheep anti-rabbit immunoglobulin.

The results of these studies showed that there was a progressive increase in mean serum PLAP-like AP levels, urinary nicotine metabolite excretion (expressed as NM/C ratios), and serum cotinine levels with increasing self-reported cigarette consumption (Table I). Thus there were highly significant and positive correlations between any two of these four variables (Table II). Similar correlations were demonstrated for biochemically proven smokers. These were also significant correlations between serum PLAP-like AP levels and both urinary nicotine metabolite excretion and serum cotinine levels among women reported to be smoking either 1–10 or 11–20 cigarettes per day (Table I).

The results presented in Table I show that there was a marked tendency for the urinary NM/C ratios and serum cotinine concentrations to plateau out with increasing numbers of cigarettes reported to have been smoked. This accords with previous evidence concerning the reduced efficiency with which cigarettes are smoked when daily consumption is very high (Feyerabend *et al.*, 1982; Peach *et al.*, 1985).

There was a strikingly high correlation ($r=0.83$) between the urinary NM/C ratios and serum cotinine levels of the 55 proven smokers in whom both estimations were made. Since the inherent errors of the urinary and serum methods are essentially independent, it was concluded that each method must provide a reliable estimate of the relative daily intake of nicotine by smokers.

Since the tar/nicotine ratios of most brands of cigarettes are very similar, both measures of nicotine intake should provide good evidence of the relative rates at which cigarette-derived carcinogens were being trapped in the lungs of different smokers, although there might be substantial individual differences in the extent to which carcinogens were activated by hepatic metabolism (Idle *et al.*, 1981).

The marked individual differences in the amounts of nicotine inhaled by smokers in each of the three self-reporting categories (Table I) is in accordance with previous evidence for differences in the efficiency with which cigarettes are smoked (Feyerabend *et al.*, 1982; Peach *et al.*, 1985), as well as inadvertent or deliberate errors of recall regarding cigarette consumption (Sillet *et al.*, 1978; Peach *et al.*, 1986). Thus in the current study 14 (3%) of the 475 volunteers who denied smoking were unequivocally identified as smokers by the diethylthiobarbituric acid method, and had NM/C ratios averaging 7.6 which was only slightly less than the mean for the self-admitted smokers (8.4). Since many of the adverse effects of smoking are markedly dose-dependent (Butler *et al.*, 1972; Doll & Peto, 1978; Doll *et al.*, 1980; Loeb *et al.*, 1984; Hirayama, 1987), these findings emphasise the importance of using objective measurements of nicotine intake (Haddow *et al.*, 1987) in epidemiological

Correspondence: G.A. Ellard.
Received 8 December 1987; and in revised form 21 March 1988.

**Table I** Correlations between serum PLAP-like AP levels and smoke intake

| | *Reported daily cigarette consumption* | | | |
|---|---|---|---|---|
| | *0* | *1–10* | *11–20* | *>20* |
| PLAP-like AP[a] | | | | |
| Mean[b] | 0.20 | 0.40 | 0.63 | 0.77 |
| (Range ±1 s.d.) | (0.14–0.27) | (0.17–0.81) | (0.31–1.35) | (0.40–1.64) |
| Median | 0.19 | 0.35 | 0.64 | 1.0 |
| (Range; N[c]) | (0.08–2.00[g]; 485) | (0.10–2.00; 66) | (0.12–2.00; 70) | (0.17–2.00; 42) |
| Urinary NM/C[d] | | | | |
| Mean[b] | 0.86 | 3.76 | 11.3 | 12.0 |
| (Range ±1 s.d.) | (0.48–1.31) | (1.27–8.43) | (5.5–20.0) | (6.7–19.4) |
| Median | 0.10 | 3.72 | 12.7 | 11.9 |
| (Range; N) | (0.1–27.5; 475) | (0.8–13.9; 47) | (0.8–27.4; 46) | (0.8–25.8; 24) |
| Serum continine[e] | | | | |
| Mean[b] | <5[h] | 25 | 108 | 127 |
| (Range ±1 s.d.) | — | (6–100) | (45–233) | (62–260) |
| Median | <5 | 29 | 117 | 163 |
| (Range; N) | (All <5; 43) | (11–276; 32) | (7–435; 51) | (13–362; 34) |
| *Correlations* | | | | |
| PLAP-like AP *vs.* urinary NM/C | | | | |
| R[f] | — | 0.47 | 0.48 | 0.40 |
| N | — | 40 | 44 | 20 |
| P | — | <0.01 | <0.001 | NS (<0.10) |
| PLAP-like AP *vs.* serum cotinine | | | | |
| R | — | 0.43 | 0.45 | 0.28 |
| N | — | 32 | 51 | 34 |
| P | — | <0.05 | <0.001 | NS (>0.10) |

[a]Optical density units; for conversion to International Units, see Tucker *et al.* (1985*b*); [b]Geometric mean; [c]Number of observations; [d]Urinary nicotine metabolites/creatinine ($\mu g\, mg^{-1}$); [e]Serum cotinine ($ng\, ml^{-1}$); [f]Linear correlation coefficient; [g]2.00 is the upper limit of the assay. There were 1, 3, 4 and 3 sera with such values within the smoking categories 0, 1–10, 11–20 and >20 cigs/day, respectively; [h]Lower limit of sensitivity.

**Table II** Correlations between serum PLAP-like AP levels and nicotine intake in self-reported smokers

| | *Serum PLAP (optical density units)* | *Serum cotinine ($ng\, ml^{-1}$)* | *Urinary nicotine metabolite ratio* |
|---|---|---|---|
| Cigarette consumption[b] | 0.32 (178)[a] | 0.56 (117) | 0.51 (117) |
| Serum PLAP-like AP (optical density units) | — | 0.49 (117) | 0.50 (104) |
| Serum cotinine ($ng\, ml^{-1}$) | — | — | 0.82 (54) |

[a]Expressed as correlation coefficient (number); [b]In calculating the correlation coefficients cigarette consumption of 1–10, 11–20 and >20 cigarettes/day have been taken as an average of 5, 15 and 25 cigarettes/day, respectively. All correlations were significant at $P<0.001$.

studies of smoking. In view of the convenience of collecting urine samples and the simplicity of the colorimetric methods for the estimation of nicotine metabolites/creatinine ratios, the urinary method would appear to be ideal for such a purpose, especially if automated modifications for their assay are employed (Barlow *et al.*, 1987; Puhakainen *et al.*, 1988).

It is probable that PLAP-like AP elevations are due to tar, or other smoke components, rather than to nicotine since we have observed that in nine smokers who were switched to chewing nicotine-impregnated gum, serum PLAP-like AP fell, after 2 months, to levels approaching those of non-smokers. The potential value of using PLAP-like AP estimations as a 'marker' for tar intake should therefore be explored, especially in investigations as to whether nicotine-enhanced cigarettes might be less hazardous than standard brands (Russell, 1976; Holland *et al.*, 1986).

The authors are indebted to the ladies of Guernsey for volunteering to participate in the prospective study. R.D. Barlow and R.B. Stone are also grateful for support from the Tobacco Products Research Trust.

### References

BARLOW, R.D., STONE, R.B., WALD, N.J. & PUHAKAINEN, E.V.J. (1987). The direct barbituric acid assay for nicotine metabolites in urine: A simple colorimetric test for the routine assessment of smoking status and cigarette smoke intake.*Clin. Chim. Acta*, **165,** 45.

BULBROOK, R.D., MOORE, J.W., CLARK, G.M.G. & WANG, D.Y. (1986). Guernsey prospective study: Relation between risk of breast cancer and biological availability of blood estradiol. *Ann. New York Acad. Sci.*, **464,** 378.

DOLL, R. (1984). Smoking and death rates. *J.A.M.A.*, **251,** 2854.

DOLL, R., GRAY, R., HAFFNER, B. & PETO, R. (1980). Mortality in relation to smoking: 22 year observations on female British doctors. *Br. Med. J.*, **i,** 967.

DOLL, R. & PETO, R. (1978). Cigarette smoking and bronchial carcinoma: Dose and time relationships among regular smokers and lifelong non-smokers. *J. Epidemiol. Commun. Hlth*, **32,** 303.

FEYERABEND, C., HIGENBOTTAM, T. & RUSSELL, M.A.H. (1982). Nicotine concentrations in urine and saliva of smokers and non-smokers. *Br. Med. J.*, **i**, 1002.

HADDOW, J.E., KNIGHT, G.J., PALOMAKI, G.E., KLOZA, E.M. & WALD, N. (1987). Cigarette consumption and serum cotinine in relation to birth weight. *Br. J. Obst. Gynaecol.*, **94**, 678.

HIRAYAMA, T. (1987). The problem of smoking and lung cancer in Japan with special reference to the rising trend in age-specific mortality rate by number of cigarettes smoked daily. *Gann*, **78**, 203.

HOLLAND, W.W., COLLEY, J.R.T. & NORTH, F. (1986). Low-tar cigarettes put to the test. *Lancet*, **ii**, 156.

IDLE, J.R., MAHGOUB, A., SLOAN, T.P., SMITH, R.L., MBANEFO, C.O. & BABABUNMI, E.A. (1981). Some observations on the oxidation phenotype status of Nigerian patients presenting with cancer. *Cancer Letters*, **11**, 331.

KNIGHT, G.J., WYLIE, P., HOLMAN, M.S. & HADDOW, J.E. (1985). Improved $^{125}$I-radioimmunoassay for continine by selective removal of bridge antibodies. *Clin. Chem.*, **31**, 118.

LOEB, L.A., ERNSTER, V.L., WARNER, K.E., ABBOTS, J. & LASZLO, J. (1984). Smoking and lung cancer: An overview. *Cancer Res.*, **44**, 5940.

MASLOW, W.C., MUENSCH, H.A., AZAMA, F. & SCHNEIDER, A.S. (1983). Sensitive fluorometry of heat-stable alkaline phosphatase (Regan enzyme) activity in serum from smokers and non-smokers. *Clin. Chem.*, **29**, 260.

McLAUGHLIN, P.J., TRAVERS, P.J., McDICKEN, I.W. & JOHNSON, P.M. (1984). Demonstration of placental and placental-like alkaline phosphatase in non-malignant human tissue extract using monoclonal antibodies in an enzyme immunoassay. *Clin. Chim. Acta*, **137**, 341.

PEACH, H., ELLARD, G.A., JENNER, P.J. & MORRIS, R.W. (1985). A simple, inexpensive urine test of smoking. *Thorax*, **40**, 351.

PEACH, H., SHAH, D. & MORRIS, R.W. (1986). Validity of smokers' information about present and past cigarette brands – implications for studies of the effect of past cigarette falling tar yields of cigarettes on health. *Thorax*, **41**, 203.

PUHAKAINEN, E.V.J., BARLOW, R.D. & SALONEN, J.T. (1987). An automated colorimetric assay for urine nicotine metabolites; a suitable alternative to continine assays for the assessment of smoking status. *Clin. Chim. Acta*, **170**, 255.

RUSSELL, M.A.H. (1976). Low-tar, medium-nicotine cigarettes: A new approach to safer smoking. *Br. Med. J.*, **i**, 1430.

SILLETT, R.W., WILSON, M.B., MALCOLM, R.E. & BALL, K.P. (1978). Deception among smokers. *Br. Med. J.*, **ii**, 1185.

TONIK, S.E., ORTMEYER, A.E., SHINDELMAN, J.E. & SUSSMAN, H.H. (1983). Elevation of serum placental alkaline phosphatase levels in cigarette smokers. *Int. J. Cancer*, **31**, 51.

TUCKER, D.F., OLIVER, R.T.D., ELLARD, G.A. & WANG, D.Y. (1985*a*). Testicular tumour marker applications for monoclonal antibodies to placental-like alkaline phosphatase. *Advances Biosciences*, **55**, 139.

TUCKER, D.F., OLIVER, R.T.D., TRAVERS, P. & BODMER, W.F. (1985*b*). Serum marker potential of placental alkaline-phosphatase-like activity in testicular germ cell tumours evaluated by H17E2 monoclonal antibody assay. *Br. J. Cancer*, **51**, 631.

WILLIAMS, G.H., McLAUGHLIN, P.J. & JOHNSON, P.M. (1986). Tissue origin of serum placental-like alkaline phosphatase in cigarette smokers. *Clin. Chem. Acta*, **155**, 329.